# Inhibition of intervertebral disc disease progression via the circPKNOX1–miR-370-3p–*KIAA0355* axis

**DOI:** 10.1038/s41420-021-00420-4

**Published:** 2021-02-26

**Authors:** Yizhen Huang, Jun Gao, Jianle Wang, Huali Ye, Teng Yao, Yining Xu, Zizheng Chen, Shuying Shen, Jianjun Ma

**Affiliations:** 1 Department of Orthopaedic Surgery, Sir Run Run Shaw Hospital, Zhejiang University School of Medicine, Zhejiang, China; 2 Key Laboratory of Musculoskeletal System Degeneration and Regeneration Translational Research of Zhejiang Province, Zhejiang, China; 3 Shaoxing University School of Medicine, Shaoxing, China

**Keywords:** Gene expression, Trauma

## Abstract

The molecular mechanism underlying the development of intervertebral disc disease (IVDD) is not completely understood. Circular RNAs (circRNAs) play a significant role in the occurrence and development of various diseases, and studies have shown that circPKNOX1 is involved in the compensatory response of extracellular matrix synthesis and secretion of the nucleus pulposus (NP) cells. However, the mechanism through which circRNAs regulate IVDD progression remains unclear; therefore, in this study, we explored the significance of circPKNOX1 in IVDD. The expression of circRNAs in NP cells of normal and degenerative patients was detected using microarray analysis, and the role of circPKNOX1 in IVDD was confirmed using RT-qPCR. The interaction networks of circRNAs, miRNAs, and miRNA target genes were detected using bioinformatics analysis, RNA fluorescence in situ hybridization, and immunofluorescence analysis. We found that the expression of circPKNOX1 decreased in IVDD cells. The expression of circPKNOX1 in NP cells, observed using RT-qPCR and western blotting, was consistent with that observed using array screening. Overexpression of circPKNOX1 increased the expression of collagen II, aggrecan, and SOX9 and decreased that of ADAMTS4, ADAMTS-5, MMP3, and MMP13. We further demonstrated that circPKNOX1 played the role of a sponge by competitively binding miR-370-3p to reverse the inhibition of *KIAA0355* expression. Our findings indicated that circPKNOX1 affected the progression of IVDD by regulating the expression of *KIAA0355* via miR-370-3p. Therefore, circPKNOX1-based therapy may serve as an effective IVDD treatment strategy.

## Introduction

Degenerative diseases of the intervertebral disc, such as intervertebral disc disease (IVDD), encompass the physiological and pathological processes of natural aging and degeneration^1^, which can cause a series of clinical symptoms, such as waist pain and numbness of the lower limbs. Serious degenerative diseases of the intervertebral disc can cause dysphoria and sexual dysfunction^2^ and affect the ability to work and quality of life. Commonly used drugs to treat these diseases include nonsteroidal anti-inflammatory drugs^3^ and opioid analgesics^4^, but these only temporarily alleviate symptoms, and the pathogenesis of IVDD remains unclear. Therefore, it is necessary to gain a more in-depth understanding of the molecular mechanism underlying the development of IVDD^5^.

There is evidence to suggest that circRNAs could be involved in miRNA inhibition and gene expression regulation, owing to which they may serve as potential therapeutic targets for IVDD^6^. circRNAs are noncoding RNA molecules with a closed ring structure, lacking a 5′ cap and 3′ poly (A) tail^7^. They are mainly located in the cytoplasm or exosomes and are not affected by RNA exonuclease^8^. circRNAs are stable, difficult to degrade, and have been shown to exist in many eukaryotes^9^. Presently, circRNAs are considered closely associated with the occurrence and development of various diseases^10^.

For example, the expression of circNR3C1 is continuously upregulated in response to the differentiation of atrophic and retinal pigment epithelium in age-related macular degeneration and is downregulated in the retinal pigment epithelium and serum of patients with dysfunctional age-related macular degeneration^11^. In addition, a previous study has shown that circNR3C1 can block the interaction between miR-382-5p and PTEN, subsequently blocking the Akt/mTOR pathway, preventing the progression of age-related macular degeneration, and protecting the retinal pigment epithelium^11^. Furthermore, in bone marrow mesenchymal stem cells with steroid-induced osteonecrosis of the femoral head, the expression of circCDR1as is upregulated^12^. Bioinformatics analysis has shown that circCDR1as plays an important role in the adipogenic/osteogenic differentiation of bone marrow mesenchymal stem cells with steroid-induced osteonecrosis of the femoral head through the CDR1as-miR-7-5p-WNT5B axis; therefore, circCDR1as may serve as a new biomarker for the diagnosis and treatment of steroid-induced osteonecrosis of the femoral head^13^.

MicroRNAs (miRNAs) are endogenous RNAs with 20–24 nucleotides^14^. In prostate cancer, the level of miR-142-3p is negatively correlated with that of the FOXO1 protein. miR-142-3p directly inhibits *FOXO1* expression by targeting its 3′-untranslated region. In addition, the inhibition of miR-142-3p impedes cell proliferation and induces cell cycle arrest, indicating that miR-142-3p gene knockout inhibits tumor growth^15^. In endometriosis, the transfection of either miR-202-3p mimic or inhibitor significantly decreases or increases the expression of *ROCK1*, respectively. Inhibition of miR-202-3p in embryonic stem cells at least partially enhances cell viability, invasion, and migration through the upregulation of *ROCK1*, leading to the occurrence of endometriosis^16^.

miRNAs have attracted attention as target gene-regulating tools for IVDD treatment^17^, and as mentioned earlier, circRNAs have been associated with various diseases. However, it remains unclear how circRNAs regulate the progress of IVDD. circPKNOX1, also termed hsa_circ_0061853 in CircBase (http://www.circbase.org), is derived from the PBX/Knotted 1 Homeobox 1 (*PKNOX1*) gene^18^. This study aimed to explore the significance and mechanism of abnormal circPKNOX1 expression in IVDD by systemically investigating its role in human cells and animal models. We also investigated the relationship between miR-370-3p and *KIAA0355*, a protein-coding gene whose product can serve as the effector of RAC1^19^ and participate in the formation of the CCR4-NOT complex and contribute to various cell activities^20^.

## Results

### Expression of circPKNOX1 decreased in degenerative discs

**Fig. 1:**
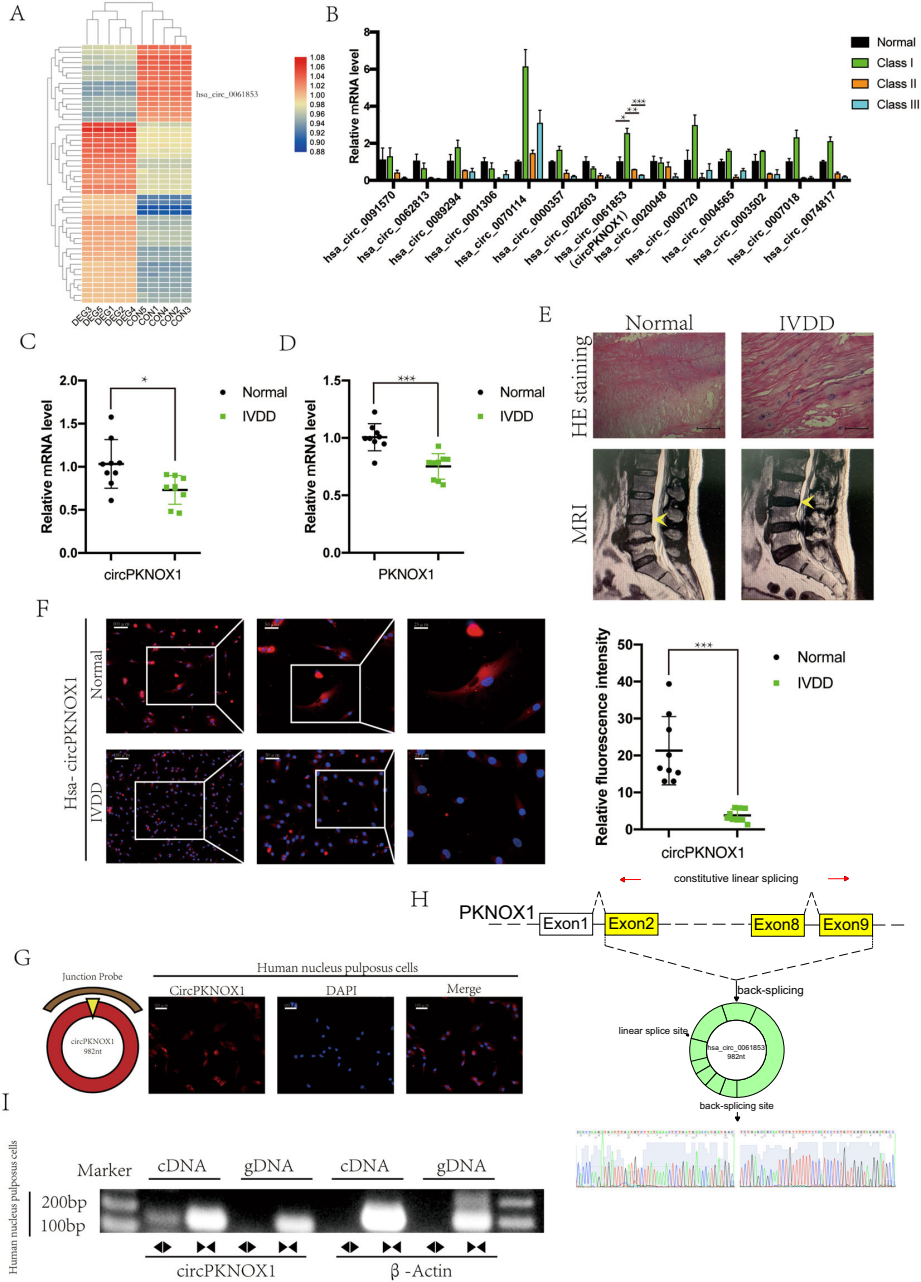
Expression of circPKNOX1 decreased in degenerative discs. **A** Heatmap of the circRNA expression matrix of degenerated tissue and control tissue. **B** Fourteen circRNAs with significantly reduced expression in degenerative tissue were selected. The expression of hsa_circ_0061853 (circPKNOX1) decreased with the severity of degeneration; *P<0.05, **P<0.01, ***P<0.001. Data represent mean±SD, and the *P* values were determined by a two-tailed unpaired Student’s *t* test. **C** The expression level of circPKNOX1 in human degenerated tissue was lower than that in control tissue; *n*=6 different donors; *P<0.05. Data represent mean±SD, and the *P* values were determined by a two-tailed unpaired Student’s *t* test. **D** The expression level of *PKNOX1* in human degenerated tissue was lower than that in control tissue; *n*=6 different donors; ***P<0.001. Data represent mean±SD, and the *P* values were determined by a two-tailed unpaired Student’s *t* test. **E** The degenerated and control tissues were analyzed using hematoxylin and eosin staining (top) and magnetic resonance imaging (MRI) (bottom). **F** Left: circPKNOX1 was labeled with a probe. The fluorescence intensity in normal tissue was higher than that in degenerated tissue (scale bar, 25–100μm); *n*=2 different donors. Right: circPKNOX1 fluorescence intensity was quantitatively analyzed; ***P<0.001. Data represent mean±SD, and the *P* values were determined by a two-tailed unpaired Student’s *t* test. **G** Left: schematic diagram of circPKNOX1 and probe targeting. Right: the circPKNOX1 probe was labeled with Cy3, and the nucleus was labeled with DAPI. CircPKNOX1 was mainly present in the cytoplasm (scale bar, 100μm). **H** Sanger sequencing confirmed th at exons 2–9 (red arrow) of *PKNOX1* composed circPKNOX1. The black arrow indicates the splice site. **I** Divergent and convergent primers of circPKNOX1 were designed. In gDNA, divergent primers could not amplify the band. β-actin served as a negative control.

We analyzed microchip data collected from previous research on circRNA and IVDD^21^. We selected 14 circRNAs (logFC > 1 or logFC < −1; *P* < 0.05) that exhibited significant changes in expression in normal and degenerated tissues (Fig. 1A). Based on the Pfirrmann classification^22^, we selected normal fracture tissues and three increased degeneration tissues (class I to class III) as the research objects and studied the changes in circRNA expression. The expression of hsa_circ_0061853, hsa_circ_0000357, hsa_circ_0000720, hsa_circ_0007018, and hsa_circ_0074817 decreased with increased disease degree. In further screening, the expression of hsa_circ_0061853 (circPKNOX1) was found to be most significantly decreased (Supplementary Fig. S3). Therefore, we speculated that circPKNOX1 might be related to the development of IVDD (Fig. 1B).

We selected three pairs of samples and tested the expression of circPKNOX1 and PKNOX1, both of which declined (Fig. 1C, D). The images of normal and degenerated tissue indicated that the intervertebral disc lost water. Hematoxylin and eosin staining indicated that the NP cells aggregated to form cell nodules in degenerated tissue (Fig. 1E). We designed a probe for circPKNOX1 and analyzed the fluorescence intensity using RNA FISH^23^. The fluorescence intensity of circPKNOX1 decreased significantly in degenerated tissues (Fig. 1F). circPKNOX1 was mainly distributed in the cytoplasm (Fig. 1G).

We then performed Sanger sequencing analysis of the circPKNOX1 junction, which indicated that circPKNOX1 was composed of *PKNOX1* exons 2–9 (Fig. 1H). To avoid trans-splicing^24^, we designed convergent and divergent primers for circPKNOX1. cDNA sequencing analysis results showed that circPKNOX1 formed multiple bands, whereas only a single band was observed in the results of gDNA sequencing analysis (Fig. 1I). The experimental results showed that circPKNOX1 was closely related to intervertebral disc degeneration and that it formed a stable circular structure in the cytoplasm.

### CircPKNOX1 delayed the process of disc degeneration in vitro

**Fig. 2:**
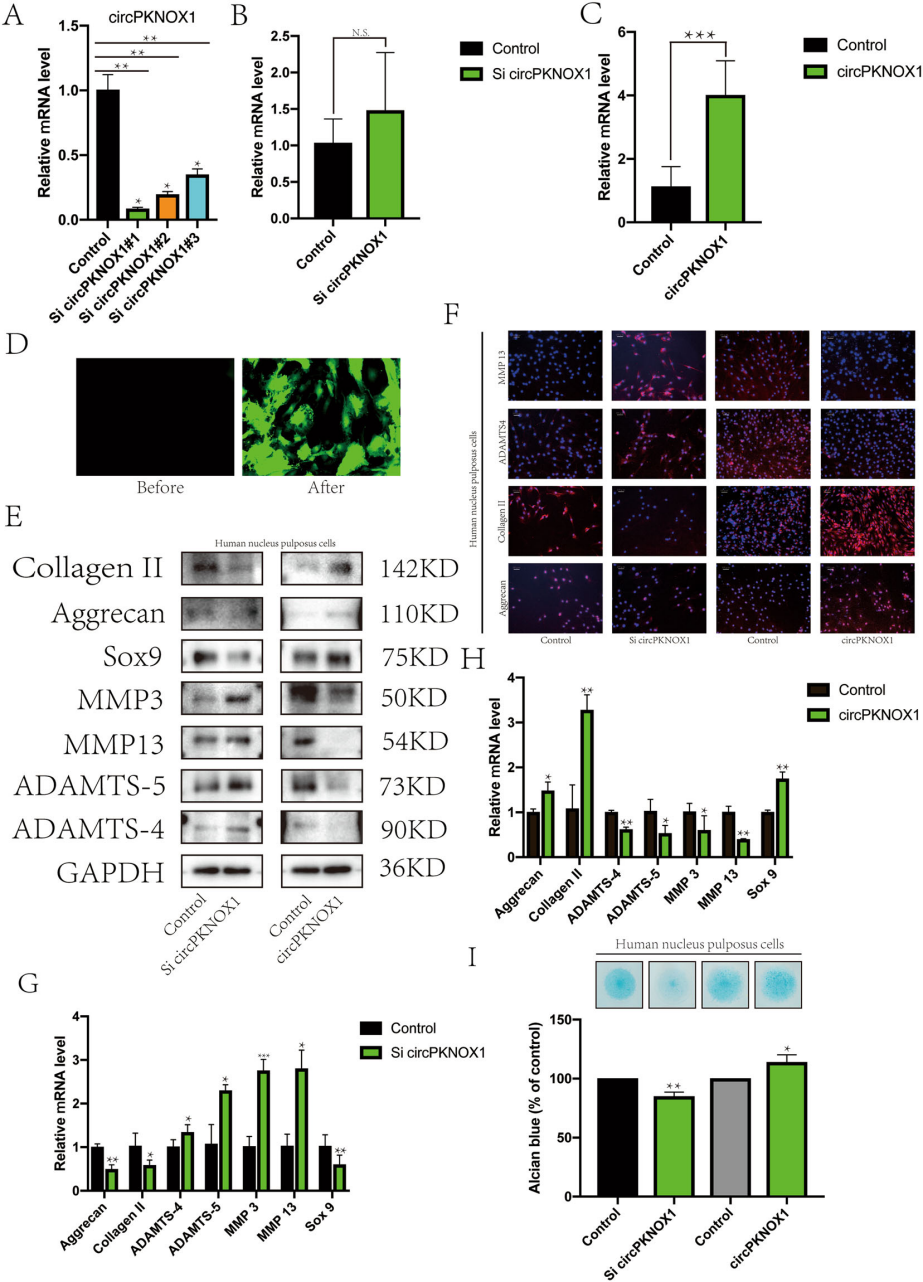
CircPKNOX1 delayed the process of disc degeneration in vitro. **A** RT-qPCR was used to detect the transfection efficiency of circPKNOX1 siRNA #1, 2, and 3; **P<0.01. Data represent mean ±SD, and the *P* values were determined by a two-tailed unpaired Student’s *t* test. **B** After knocking down circPKNOX1, the expression level of PKNOX1 was detected. Data represent mean±SD, and the *P* values were determined by a two-tailed unpaired Student’s *t* test. **C** Adenoviruses containing circPKNOX1 were used to infect nucleus pulposus cells, and the infection efficiency was detected; ***P<0.001. Data represent mean±SD, and the *P* values were determined by a two-tailed unpaired Student’s *t* test. **D** Green fluorescence indicates infected nucleus pulposus cells. **E** After circPKNOX1 was knocked down or overexpressed, the expression levels of related proteins (collagen II, aggrecan, SOX9, MMP3, MMP13, ADAMTS4, and ADAMTS-5) were detected using western blotting. **F** The fluorescence intensity of related proteins (collagen II, aggrecan, ADAMTS4, and MMP13) was observed using an immunofluorescence assay (scale bar, 100μm). After circPKNOX1 was knocked down (**G**) or circPKNOX1 was overexpressed (**H**), RT-qPCR was used to detect the expression of related genes; *P<0.05, **P<0.01. Data represent mean±SD, and the *P* values were determined by a two-tailed unpaired Student’s *t* test. **I** Top: the extracellular matrix was detected by Alcian staining. Bottom: the Alcian staining was quantitatively analyzed. Data represent mean±SD, and the *P* values were determined by a two-tailed unpaired Student’s *t* test.

Next, we examined the effects of inhibiting and overexpressing circPKNOX1. We designed three types of siRNA and chose the most suitable one for the experiment (Fig. 2A). After inhibiting circPKNOX1, *PKNOX1* expression did not change significantly (Fig. 2B). We used adenovirus as a vector to introduce and overexpress circPKNOX1, and the fluorescence intensity and RT-qPCR results showed that the expression of circPKNOX1 increased significantly (Fig. 2C, D).

circPKNOX1 promoted matrix synthesis and inhibited the decomposition of corresponding proteases. When circPKNOX1 expression was suppressed, this trend disappeared (Fig. 2E). The results of the IF assay^25^ and RT-qPCR also supported this finding (Fig. 2F–H). The Alcian staining results showed that circPKNOX1 affected matrix synthesis and decomposition (Fig. 2I). Hence, we concluded that circPKNOX1 promoted the activity of NP cells and inhibited IVDD.

### circPKNOX1 acted as a sponge for miR-370-3p in NP cells

**Fig. 3:**
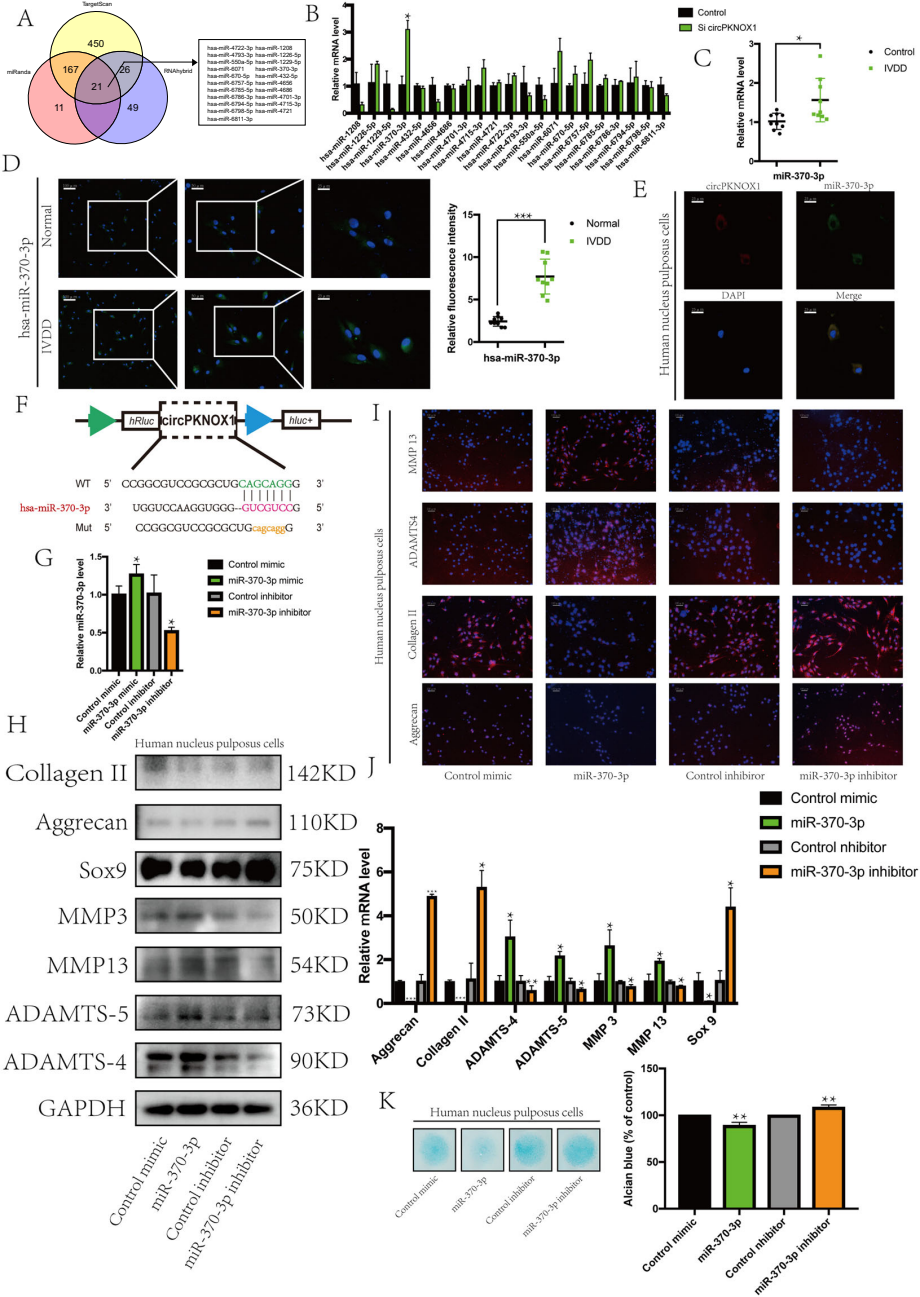
circPKNOX1 acted as a sponge for miR-370-3p in NP cells. **A** Through the intersection of TargetScan, miRanda, and RNA hybrid data, circPKNOX1 target microRNA was predicted. **B** After knocking down circPKNOX1, the expression level of hsa-miR-370-3p increased; *P<0.05. Data represent mean±SD, and the *P* values were determined by a two-tailed unpaired Student’s *t* test. **C** The expression level of miR-370-3p increased in degenerated tissues (six different donors); *P<0.05. Data represent mean±SD, and the *P* values were determined by a two-tailed unpaired Student’s *t* test. **D** Left: the fluorescence intensity of miR-370-3p increased in degenerated tissues, as determined using fluorescence in situ hybridization (scale bar, 25–100μm). Right: the fluorescence intensity was quantitatively analyzed (two different donors); ***P<0.01. Data represent mean±SD, and the *P* values were determined by a two-tailed unpaired Student’s *t* test. The miR-370-3p probe was labeled with Alexa Fluor 488. **E** miR-370-3p was located in the cytoplasm and intersected with circPKNOX1 in the cell substructure (scale bar, 25μm). **F** The binding site of circPKNOX1 and miR-370-3p was identified using CircInteractome. **G** The efficiency of miR-370-3p mimic and inhibitor was detected; *P<0.05. Data represent mean±SD, and the *P* values were determined by a two-tailed unpaired Student’s *t* test. **H** The expression of related proteins (collagen II, aggrecan, SOX9, MMP3, MMP13, ADAMTS4, and ADAMTS-5) was detected using western blotting. **I** The fluorescence intensity of related proteins (collagen II, aggrecan, ADAMTS4, and MMP13) was detected via an immunofluorescence assay (scale bar, 100μm). **J** RT-qPCR was used to detect the expression of related genes; *P<0.05, **P<0.01, ***P<0.001. Data represent mean±SD, and the *P* values were determined by a two-tailed unpaired Student’s *t* test. **K** Left: the extracellular matrix was detected by Alcian staining. Right: the Alcian staining was quantitatively analyzed. Data represent mean±SD, and the *P* values were determined by a two-tailed unpaired Student’s *t* test.

**Fig. 4:**
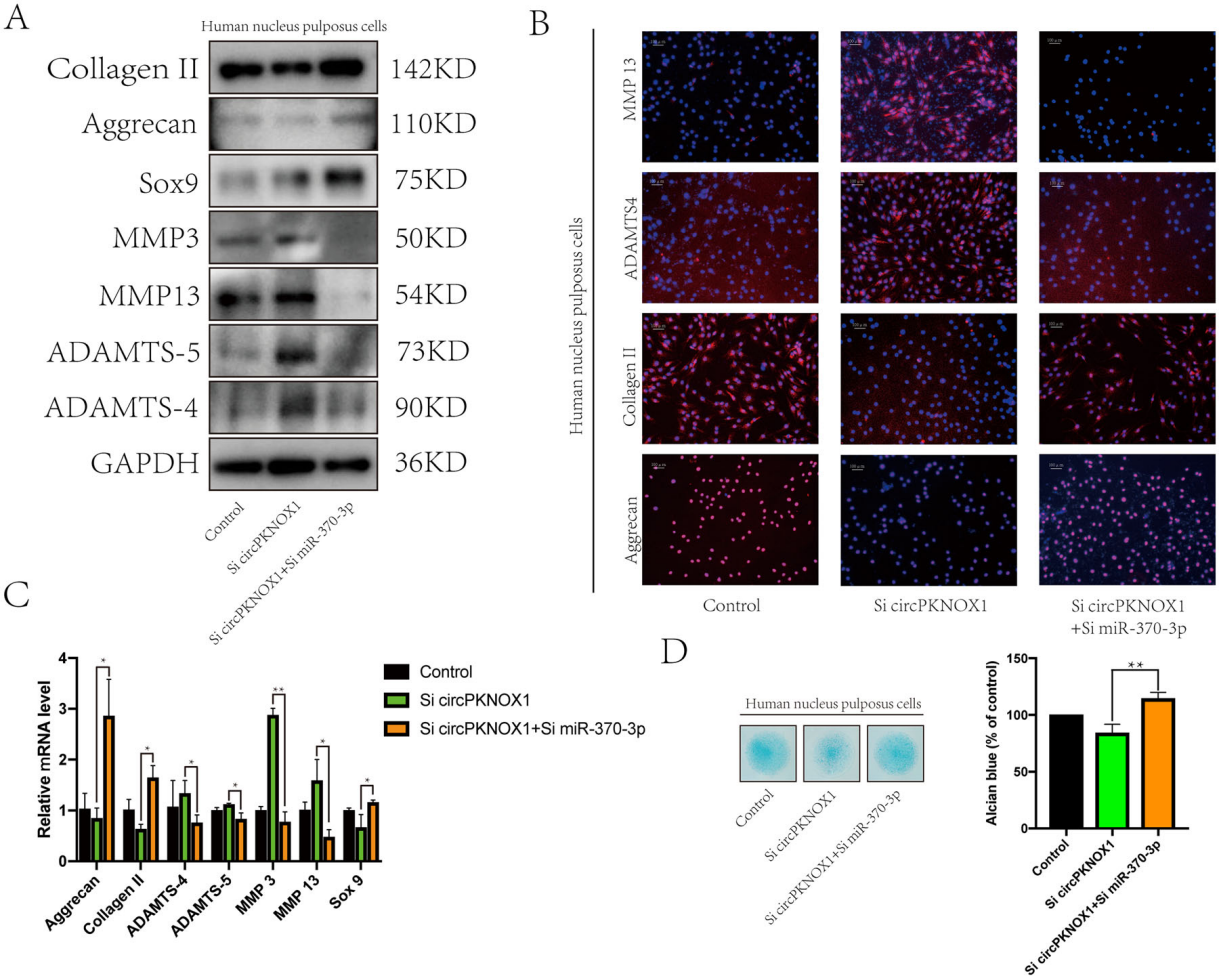
circPKNOX1 regulated NP cells via miR-370-3p. **A** Expression of related proteins (collagen II, aggrecan, SOX9, MMP3, MMP13, ADAMTS4, and ADAMTS-5) was reversed. **B** The fluorescence intensity of related proteins (collagen II, aggrecan, ADAMTS4, and MMP13) was observed via an immunofluorescence assay (scale bar, 100μm). **C** RT-qPCR was used to detect the expression of related genes (collagen II, aggrecan, *SOX9*, *MMP3*, *MMP13*, *ADAMTS4*, and *ADAMTS5*); *P<0.05, **P<0.01. Data represent mean±SD, and the *P* values were determined by a two-tailed unpaired Student’s *t* test. **D** Left: the extracellular matrix was detected by Alcian staining. Right: the Alcian staining was quantitatively analyzed. Data represent mean±SD, and the *P* values were determined by a two-tailed unpaired Student’s *t* test.

We used TargetScan, miRanda, and RNAhybrid to predict the downstream miRNAs of circPKNOX1 and obtained 21 miRNAs from the intersection of the results (Figs. 3A and 4). We then knocked down circPKNOX1 in NP cells and detected the expression of the 21 miRNAs. The expression of miR-370-3p significantly increased (Fig. 3B); therefore, we speculated that miR-370-3p was related to disc degeneration^26^. Furthermore, the results of RT-qPCR indicated that miR-370-3p expression increased in degenerated tissues (Fig. 3C). We designed a probe for miR-370-3p and found that the fluorescence intensity in degenerated tissue was stronger than that in normal tissue (Fig. 3D). We marked circPKNOX1 and miR-370-3p in the NP cells; both were present in the cytoplasm and spatially consistent (Fig. 3E). The results of the online software analysis of the circPKNOX1 and miR-370-3p binding sites are shown in Fig. 3F.

To further explore the biological function of miR-370-3p, we inhibited and overexpressed miR-370-3p in NP cells (Fig. 3G). In addition, based on the results of western blotting, IF, and RT-qPCR, we concluded that miR-370-3p inhibited the expression of collagen II, aggrecan, and SOX9 but promoted the expression of ADAMTS4, ADAMTS-5, MMP3, and MMP13. After knocking down miR-370-3p, the opposite result was obtained (Fig. 3H–J). miR-370-3p affected the extracellular matrix of NP cells, which was also confirmed by Alcian staining results (Fig. 3K). In summary, as a downstream miRNA of circPKNOX1, miR-370-3p had a negative effect on NP cells.

### circPKNOX1 regulated NP cells via miR-370-3p

To verify that circPKNOX1 and miR-370-3p were related to biological functions, we designed a rescue experiment^27^. In one group, we knocked down circPKNOX1 alone, and in the other group, we knocked down circPKNOX1 and miR-370-3p together. After knocking down circPKNOX1 alone, the expression levels of collagen II, aggrecan, and SOX9 decreased, while those of ADAMTS4, ADAMTS-5, MMP3, and MMP13 increased. When circPKNOX1 and miR-370-3p were both knocked down, the expression levels of collagen II, aggrecan, and SOX9 increased, while those of ADAMTS4, ADAMTS-5, MMP3, and MMP13 decreased (Fig. 4A–C). Furthermore, this trend was also reflected by the Alcian staining results (Fig. 4D). This evidence suggested that miR-370-3p was a downstream molecule of circPKNOX1.

### circPKNOX1 retarded the IVDD process via the miR-370-3p/*KIAA0355* axis

**Fig. 5:**
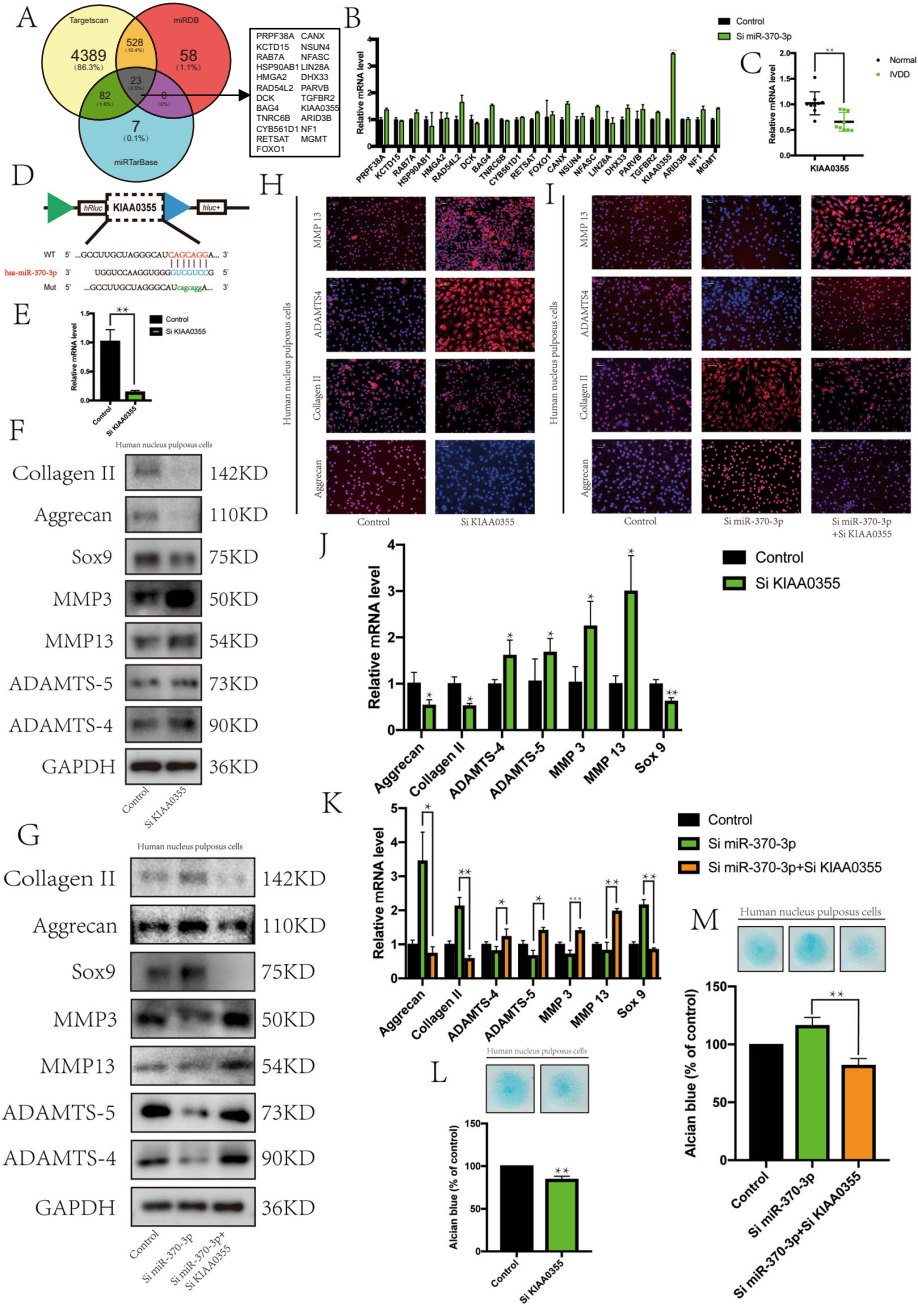
circPKNOX1 retarded the IVDD process via the miR-370-3p/ KIAA0355 axis. **A** miR-370-3p target genes were predicted using TargetScan, miRDB, and miRTarBase. **B** After knocking down miR-370-3p, the expression level of *KIAA0355* increased; ***P<0.001. Data represent mean±SD, and the *P* values were determined by a two-tailed unpaired Student’s *t* test. **C** In degenerated tissues, the expression of *KIAA0355* decreased; **P<0.01. Data represent mean±SD, and the *P* values were determined by a two-tailed unpaired Student’s *t* test. **D** The binding site of miR-370-3p and *KIAA0355* was predicted using TargetScan. **E** After transfection with siKIAA0355, the expression of *KIAA0355* decreased; **P<0.01. Data represent mean±SD, and the *P* values were determined by a two-tailed unpaired Student’s *t* test. The expression of related proteins (collagen II, aggrecan, SOX9, MMP3, MMP13, ADAMTS4, and ADAMTS-5) was observed by western blotting (**F**) after *KIAA0355* knockdown and (**G**) after *KIAA0355* and miR-370-3p knockdown. The fluorescence intensity of related proteins (collagen II, aggrecan, ADAMTS4, and MMP13) (scale bar, 100μm) was observed via an immunofluorescence assay (**H**) after *KIAA0355* knockdown and (**I**) after *KIAA0355* and miR-370-3p knockdown. RT-qPCR was used to detect the expression levels of related genes (**J**) after *KIAA0355* knockdown (*P<0.05, **P<0.01. Data represent mean±SD, and the *P* values were determined by a two-tailed unpaired Student’s *t* test) and (**K**) after *KIAA0355* and miR-370-3p knockdown (*P<0.05, **P<0.01, ***P<0.001. Data represent mean±SD, and the *P* values were determined by a two-tailed unpaired Student’s *t* test.). Top: the extracellular matrix was detected by Alcian staining (**L**) after *KIAA0355* knockdown and (**M**) after *KIAA0355* and miR-370-3p knockdown. Bottom: the Alcian staining was quantitatively analyzed. Data represent mean±SD, and the *P* values were determined by a two-tailed unpaired Student’s *t* test.

We used TargetScan, miRDB, and miRTarBase to predict the downstream target genes of miR-370-3p and obtained 23 possible target genes (Fig. 5A). To identify the most suitable target genes, we tested the expression of these target genes when miR-370-3p was knocked down. Among these target genes, the expression of *KIAA0355* was significantly increased, and we speculated that *KIAA0355* might be a downstream target gene of miR-370-3p^20^ (Fig. 5B). Compared with that in normal tissues, the expression of *KIAA0355* decreased significantly in degenerated tissues (Fig. 5C). Through TargetScan, we also identified the binding site of miR-370-3p and *KIAA0355* (Fig. 5D).

Next, we explored whether *KIAA0355* and IVDD were related and tested the efficiency of *KIAA0355* siRNA (Fig. 5E). The knockdown of *KIAA0355* was associated with a decrease in the expression of collagen II, aggrecan, and SOX9 and an increase in the expression of ADAMTS4, ADAMTS-5, MMP3, and MMP13 (Fig. 5F, H, J). Moreover, when the expression of *KIAA0355* decreased, the degradation of the extracellular matrix was accelerated (Fig. 5L). Therefore, we concluded that *KIAA0355* was a target gene related to IVDD.

Finally, we evaluated the relationship between miR-370-3p and *KIAA0355* through a rescue experiment. In NP cells, we knocked down miR-370-3p either alone or with *KIAA0355*. When miR-370-3p was knocked down alone, we observed a positive effect on NP cells. The expression of collagen II, aggrecan, and SOX9 increased, and the expression of ADAMTS4, ADAMTS-5, MMP3, and MMP13 decreased. Co-knockdown of *KIAA0355* and miR-370-3p was not conducive to the growth of NP cells; the corresponding matrix synthesis was reduced, and the decomposition enzyme levels were increased (Fig. 5G, I, K). Notably, the Alcian staining results were consistent (Fig. 5M). Therefore, we concluded that circPKNOX1 regulated the growth, synthesis, and decomposition of NP cells through the miR-370-3p–*KIAA0355* axis.

### In the animal model, the circPKNOX1/miR-370-3p/*KIAA0355* axis promoted IVDD development

**Fig. 6:**
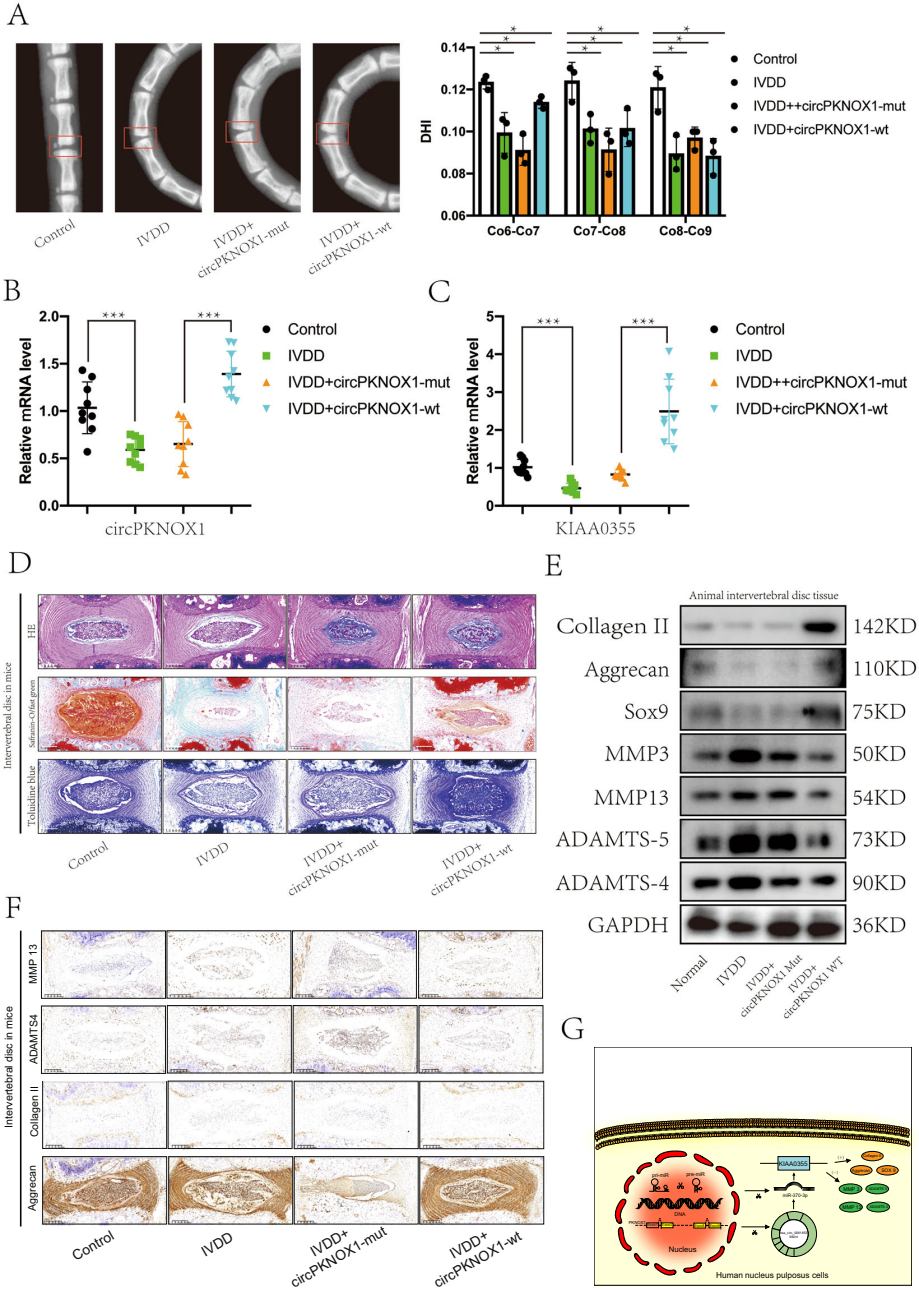
In the animal model, the circPKNOX1/miR-370-3p/ KIAA0355 axis promoted IVDD development. **A** Left: the tails of the mice were examined using X-ray imaging analysis. Right: the disc height index of the tails of the mice was quantitatively analyzed; *P<0.05. Data represent mean±SD, and the *P* values were determined by a two-tailed unpaired Student’s *t* test. **B** The expression of circPKNOX1 was detected using RT-qPCR; ***P<0.001. Data represent mean±SD, and the *P* values were determined by a two-tailed unpaired Student’s *t* test. **C** The expression of *KIAA0355* was detected using RT-qPCR; ***P<0.001. Data represent mean±SD, and the *P* values were determined by a two-tailed unpaired Student’s *t* test. **D** The intervertebral disc tissue of mice was observed using hematoxylin and eosin, Safranin-O/fast green, and toluidine blue staining (scale bar, 200μm). **E** In the control, IVDD, circPKNOX1-mut, and circPKNOX1-wt groups, the expression of related proteins (collagen II, aggrecan, SOX9, MMP3, MMP13, ADAMTS4, and ADAMTS-5) was detected using western blotting. **F** Sections of the discs were analyzed using immunohistochemical staining for collagen II, aggrecan, ADAMTS4, and MMP13 (scale bar, 200μm). **G** The mechanism of action of circPKNOX1.

The degree of bend in the tail of the model corresponded to the decrease in the disc height index (Fig. 6A). The expression of circPKNOX1 and *KIAA0355* in the intervertebral disc of the tail was increased in the circPKNOX1-wt group (Fig. 6B, C). After several staining procedures (hematoxylin and eosin, Safranin-O/fast green, and toluidine blue), we found that the NP tissue was extruded by pressure and lost (Fig. 6D). We detected the expression of related anabolic and catabolic proteins in the intervertebral discs of the mice using western blotting (Fig. 6E). Anabolism was decreased in the degenerative group, and catabolism was increased, but this trend was reversed when circPKNOX1 expression increased. Finally, immunohistochemical staining showed that the expression of collagen II, aggrecan, ADAMTS4, and MMP13 decreased compared with that in the circPKNOX1-mut and circPKNOX1-wt groups (Fig. 6F). In summary, these results demonstrated that circPKNOX1 had a therapeutic effect on intervertebral disc degeneration.

## Discussion

Although the mechanism through which circRNAs regulate the progression of IVDD is not clear, accumulating evidence indicates that circRNAs may act as miRNA sponges^28^, translating or interacting with transcription factors to regulate the transcription of their genes. Some studies have shown that circVMA21, like a sponge of miR-200c, can reduce the apoptosis of NP cells induced by inflammatory cytokines and the imbalance between extracellular matrix anabolism and catabolism through the XIAP pathway. The expression of XIAP in the NP cells is directly related to apoptosis and imbalances in the expression of anabolic and catabolic factors in the extracellular matrix. miR-200c, as an upstream molecule, regulates the activity and function of NP cells by inhibiting XIAP. As a sponge of miR-200c, circVMA21 plays a role in NP cells by targeting miR-200c and XIAP. In addition, IVDD in rats can be reduced by administering an intradiscal injection of circVMA21 in vivo^29^. The results of our bioinformatics analysis showed that circPKNOX1 had multiple potential binding sites for multiple miRNAs. The results of FISH and double-luciferase analysis showed that miR-370-3p directly combined with circPKNOX1, providing a potentially effective treatment strategy against IVDD.

In summary, circPKNOX1 regulated the expression of *KIAA0355* through the miR-370-3p pathway, thus affecting the progression of IVDD (Fig. 6G). Therefore, IVDD treatment based on circPKNOX1 may be an effective therapeutic strategy. A limitation of this study was the age gap between IVDD and non-IVDD patients, which may have introduced bias; further in vitro and in vivo experiments are needed to eliminate the influence of age. In addition, as miRNA sponges, circRNAs may perform other functions in IVDD that require further investigation. For instance, circRNAs act as dynamic scaffolds for regulating protein-protein interactions^30,31^. Advances in chemical and biological research are needed to improve the current techniques for recognizing these interactions.

## Materials and methods

### Human intervertebral disc tissue

According to the procedures of the Ethics Committee of Shaw Hospital in Zhejiang, we collected human nucleus pulposus (NP) tissue after surgery. Before the operation, all the patients, after being informed of all risks and possible outcomes, signed informed consent forms. All specimens were stored at −80 °C, according to the specifications. The control group comprised patients with lumbar vertebrae trauma (*n* = 3), and the pathological group comprised of patients with herniated discs (*n* = 49). Patient information is provided in Supplementary Table S1^32^.

### Mouse disc degeneration model

C57 mice were purchased from Shanghai SLAC Laboratory Animal Co., Ltd. (Shanghai, China). All animal experiments were conducted according to the principles and procedures of the National Institutes of Health Guide for the Care and Use of Laboratory Animals and the guidelines of Sir Run Run Shaw Hospital (Zhejiang University, Hangzhou, Zhejiang, China) for animal treatment.

After anesthesia was administered, the tails of the mice were fixed with a thin wire, which was then bent into a ring to simulate the chronic degeneration of the intervertebral disc under pressure^33^. One month after the tail was fixed, the mice were divided into four groups. Wild-type (wt) and mutant (mut) circPKNOX1 adenoviruses were constructed and provided by Hanbio Biotechnology Co., Ltd. (Shanghai, China). After successful modeling, the experimental and control groups were injected intraperitoneally with adenovirus solution and normal saline, respectively, once a week for 8 weeks. At the end of the experiment, the mice were euthanized, and the tails were removed for X-ray imaging analysis.

### NP cell culture

To extract NP cells, intervertebral disc tissue was isolated from humans and mice, and digested with collagenase type II (Sigma, USA). The NP cells were cultured at 37 °C in a humidified environment containing 5% carbon dioxide. During cultivation, nutrients were supplied by Dulbecco’s Modified Eagle Medium (DMEM) containing 10% fetal bovine serum (ThermoFisher Scientific, Waltham, MA, USA).

### Bioinformatics analysis

We predicted the downstream miRNA of circPKNOX1 using three analysis tools: TargetScan (http://www.targetscan.org/), miRanda (http://www.microrna.org/), and RNAhybrid. We used the intersection of data from three online databases, TargetScan, miRDB (http://mirdb.org), and miRTarBase (http://mirtarbase.mbc.nctu.edu.tw/php/download.php), to predict the target gene of miR-370-3p. The binding site was analyzed using TargetScan and CircInteractome (https://circinteractome.nia.nih.gov/index.html).

### RNA extraction and RT-qPCR analysis

The total cellular RNA was extracted from cultured NP cells using TRIzol reagent (Invitrogen, Carlsbad, CA, USA) and the Ultrapure RNA Kit (CWBIO, Beijing, China) according to the manufacturer’s instructions. miRNA was extracted using a miRNA extraction kit (CWBIO). After reverse transcribing RNA into cDNA using a reverse transcription kit (Accurate, Hunan, China), miRNA and mRNA expression was analyzed using RT-qPCR. The reaction mixture contained 1 µl of cDNA, 5 µl of SYBR Green Master Mix (Yeasen Biotech Co., Ltd., Shanghai, China), 1 µl of primer (TsingKe, Hangzhou, China), and 3 µl of water (10 µl total). The reaction was carried out in an ABI 7500 Sequencing Detection System (Applied Biosystems, Foster City, CA, USA) under the following conditions: 40 cycles of denaturation at 95 °C for 5 s and amplification at 60 °C for 24 s. The primer details are provided in Supplementary Table S2.

### Adenovirus overexpression

The construction and extraction of adenovirus circPKNOX1 and insertion of PCR-cloned circ gene cDNA (Ad-circPKNOX1) and green fluorescent protein gene cDNA (Ad-GFP) into a pAdEasy-EF1-MCS-CMV-GFP vector were performed by Han Biological, Shanghai, China. We infected cells with the virus and adjusted the amount of virus according to the titer and cell number (virus amount = MOI × cell number/virus titer (PFU/ml) × 1000). After 4 h of infection, the medium was changed, and the cells were observed under a fluorescence microscope to determine whether the infection was successful. Finally, the expression levels were detected using PCR^34^.

### RNA interference and overexpression

We suppressed the expression of circPKNOX1 through a siRNA-mediated gene and used specific inhibitors and mimetics (Ribobio, Guangzhou, China) to inhibit or induce the expression of miR-370-3p, respectively. We introduced siRNA into cells, using the Lipofectamine RNAiMAX transfection reagent (ThermoFisher), and detected related expression levels after 48 h.

### Alcian blue staining

Alcian staining was performed with an Alcian blue stain kit (G1563, Solarbio). NP cells were seeded onto 24-well plates and fixed with 10% neutral formalin after transfection. The cells were acidified for 5 min and then stained for 30 min. Finally, excess Alcian dye was removed, and the cells were washed and observed under the scanner (V600, EPSON).

### Western blotting

Human or mouse NP cells were lysed with a radioimmunoprecipitation assay buffer (Beyotime, China) to extract cellular proteins. The proteins were separated using 10% SDS-PAGE. The separated proteins were transferred from the gel to a polyvinylidene fluoride membrane (Bio-Rad, Hercules, CA, USA) and blocked with skimmed milk powder for 1 h. Subsequently, the membrane was immersed in the relevant protein antibody solution (collagen II, aggrecan, SOX9, MMP3, MMP13, ADAMTS4, or ADAMTS-5; 1:1000; Abcam) at 4 °C for 8 h. After washing away excess antibody, the membrane was incubated with a secondary antibody at room temperature (25 °C) for 2 h. The protein bands were observed with an Amersham lmager 600 (General Electric Company, USA) and analyzed with the ImageJ software (National Institute of Health, Bethesda, MD, USA).

### Immunofluorescence (IF) assay

The treated NP cells were fixed with 4% paraformaldehyde for 20 min. After the paraformaldehyde was washed off, the cell membrane was permeabilized with 0.3% Triton X-100 and then blocked with 5% BSA for 60 min. The cells were immersed in the relevant antibody solution (collagen II, aggrecan, MMP3, or ADAMTS-5; 1:1000; Abcam) at 4 °C for 8 h. After excess antibody was washed off, the cells were incubated in goat anti-rabbit IgG conjugated to fluorescent Cy5 dye (1:100; Abcam), for 2 h in the dark. Nuclei were stained with DAPI (Life Technologies, Carlsbad, CA, USA). A Zeiss LSM780 confocal microscope was used to observe cell fluorescence. IF images were captured using a Nikon Eclipse TI and merged using Image-Pro Plus 6.0 (National Institute of Health).

### RNA fluorescence in situ hybridization (FISH)

A Cy3-labeled circPKNOX1 probe and a 488-labeled locked nucleic acid miR-370-3p probe were designed and synthesized by HaoKe, Wuhan, China. A FISH kit (RiboBio, Guangzhou, China) was used to detect the NP cell fluorescence signal. These images were captured using the Nikon A1Si Laser Scanning Confocal Microscope (Nikon Instruments Inc., Japan).

### Statistical analysis

Statistical analysis was performed using SPSS v22.0. The unpaired data between the two groups were analyzed using a *t* test (with a 95% confidence interval for the differences between groups). Values of *P* < 0.05 were considered statistically significant.

## Supplementary information

Supplementary_Table.docx

Supplementary Figure Legends.docx

Supplementary Figure Legends.tif
