# Supplementary material for: Inhibition of intervertebral disc disease progression via the circPKNOX1–miR-370-3p–KIAA0355 axis
Source: Cell Death Discov. 2021 Feb 26;7:39. doi: 10.1038/s41420-021-00420-4 (PMC7910476; doi:10.1038/s41420-021-00420-4)
Supplement: Supplementary file 1 — Supplementary_Table.docx [file 41420_2021_420_MOESM1_ESM.docx]

***Supplementary Materials***

**Inhibition of intervertebral disc disease progression via the circPKNOX1–miR-370-3p–KIAA0355 axis**

*Yizhen Huang, Jun Gao, Jianle Wang, Huali Ye, Teng Yao, Yining Xu, Zizheng Chen, Shuying Shen, and Jianjun Ma*

**Table S1:**

**Control**

| **Number** | **Gender** | **Age (year)** | **Height (cm)** | **Weight (kg)** | **BMI** |
| --- | --- | --- | --- | --- | --- |
| 55xxx06 | Male | 33 | 170 | 78.5 | 27.16263 |
| 31xxx57 | Female | 36 | 160 | 51 | 19.92188 |
| 55xxx18 | Female | 51 | 152 | 46 | 19.90997 |

**Intervertebral disc disease**

| **Number** | **Gender** | **Age (year)** | **Height (cm)** | **Weight (kg)** | **BMI** |
| --- | --- | --- | --- | --- | --- |
| 50xxx96 | Female | 24 | 156 | 70 | 28.76397 |
| 30xxx98 | Female | 32 | 171 | 64 | 21.88708 |
| 56xxx30 | Male | 34 | 169 | 58.8 | 20.58751 |
| 55xxx50 | Female | 42 | 154 | 46 | 19.39619 |
| 55xxx97 | Male | 43 | 185 | 80 | 23.37473 |
| 55xxx95 | Female | 46 | 160 | 65.6 | 25.62500 |
| 56xxx06 | Female | 47 | 158 | 67.5 | 27.03894 |
| 46xxx90 | Female | 49 | 160 | 52.5 | 20.50781 |
| 55xxx03 | Female | 50 | 159 | 75 | 29.66655 |
| 57xxx35 | Male | 52 | 168 | 66.85 | 23.68552 |
| 56xxx11 | Male | 54 | 172 | 68 | 22.98540 |
| 53xxx50 | Female | 54 | 160 | 58.5 | 22.85156 |
| 24xxx09 | Female | 54 | 156 | 61 | 25.06575 |
| 80xxx03 | Male | 54 | 155 | 51 | 21.22789 |
| 54xxx99 | Female | 55 | 160 | 60 | 23.43750 |
| 43xxx76 | Male | 56 | 170 | 73.8 | 25.53633 |
| 56xxx01 | Male | 56 | 171 | 80 | 27.35885 |
| 29xxx98 | Female | 56 | 158 | 59 | 23.63403 |
| 56xxx56 | Male | 56 | 171 | 80 | 27.35885 |
| 56xxx20 | Male | 56 | 167 | 65 | 23.30668 |
| 28xxx94 | Female | 57 | 160 | 60 | 23.43750 |
| 18xxx67 | Female | 57 | 157 | 56 | 22.71897 |
| 81xxx38 | Female | 60 | 159 | 75 | 29.66655 |
| 56xxx00 | Male | 60 | 170 | 62 | 21.45329 |
| 56xxx62 | Female | 60 | 146 | 47.6 | 22.33064 |
| 55xxx15 | Female | 62 | 155 | 52 | 21.64412 |
| 56xxx07 | Female | 62 | 160 | 74 | 28.90625 |
| 55xxx78 | Female | 62 | 150 | 55.6 | 24.71111 |
| 56xxx16 | Male | 63 | 175 | 90 | 29.38776 |
| 36xxx62 | Male | 63 | 169 | 87 | 30.46112 |
| 56xxx90 | Female | 63 | 158 | 65 | 26.03749 |
| 56xxx35 | Male | 63 | 168 | 72 | 25.51020 |
| 56xxx88 | Male | 64 | 175 | 68 | 22.20408 |
| 56xxx43 | Male | 65 | 160 | 65.5 | 25.58594 |
| 54xxx86 | Male | 65 | 176 | 62 | 20.01550 |
| 51xxx33 | Female | 66 | 154 | 53 | 22.34778 |
| 20xxx56 | Male | 67 | 170 | 85 | 29.41176 |
| 54xxx24 | Male | 68 | 170 | 54 | 18.68512 |
| 32xxx29 | Male | 69 | 173 | 68 | 22.72044 |
| 55xxx67 | Male | 70 | 175 | 65 | 21.22449 |
| 55xxx21 | Female | 70 | 158 | 68 | 27.23922 |
| 44xxx05 | Female | 70 | 152 | 41.5 | 17.96226 |
| 36xxx09 | Female | 72 | 155 | 66.5 | 27.67950 |
| 56xxx65 | Male | 73 | 160 | 75 | 29.29688 |
| 56xxx37 | Male | 73 | 168 | 69 | 24.44728 |
| 23xxx59 | Male | 73 | 170 | 65.5 | 22.66436 |
| 80xxx68 | Female | 75 | 158 | 56 | 22.43230 |
| 19xxx68 | Male | 75 | 172 | 82 | 27.71769 |
| 55xxx44 | Male | 82 | 168 | 68 | 24.09297 |

**Table S2:**

|  | **Sequence (5ʹ–3ʹ)** |
| --- | --- |
| hsa_circ_0061853 F | GGCATCCCTACCCAACAGAG |
| hsa_circ_0061853 R | AGCGAATGGTGTCAGTTGTTG |
| hsa_circ_0091570 F | CTACACCTACCACTGTGTCTGC |
| hsa_circ_0091570 R | AAGCCATGGGAGGATTAGCTG |
| hsa_circ_0062813 F | AAGAGCAACATGGAGACCTGT |
| hsa_circ_0062813 R | CTGACACCTGATAACTTTGCAGTC |
| hsa_circ_0089294 F | AAAATTCCTACGGCTGCATGG |
| hsa_circ_0089294 R | AGCTCTCTCGTTGGCACG |
| hsa_circ_0001306F | CAGGAAGCCATCACACTCCT |
| hsa_circ_0001306 R | CCCAGCCTACATACAGTGAACC |
| hsa_circ_0070114 F | ACGGCTTCTACCAAGATCGC |
| hsa_circ_0070114 R | ACTGCTCACCATGCTCGTAT |
| hsa_circ_0000357 F | CCAGGCCTTCAAAGCTCACCT |
| hsa_circ_0000357 R | AGGGGGCCCATTCCATTCAAC |
| hsa_circ_0022603 F | TCTACAAGTCCGTCATCCAAGC |
| hsa_circ_0022603 R | TGATCAGCGTGGTGCCAAAG |
| hsa_circ_0020048 F | TAGACAAGCCCTCAAGGATGC |
| hsa_circ_0020048 R | TACTGAACTGCAATCTGGTGT |
| hsa_circ_0000720 F | TGGAGGACAAGAAGGACGAAC |
| hsa_circ_0000720 R | TTGATCTTGGTAGTCCGCGTC |
| hsa_circ_0004565 F | TTGACATTCCATCTGTCTTTATTGG |
| hsa_circ_0004565 R | TGGTGTAGACTTGTGTGGCTG |
| hsa_circ_0003502 F | CCCTTCCTTATCATAGTGGGCAT |
| hsa_circ_0003502 R | ATGGTGTAGACTTGTGTGGCT |
| hsa_circ_0007018 F | TCCATTTCCTCCCACGAATG |
| hsa_circ_0007018 R | ACTTCCACATGGGGAGATACAG |
| hsa_circ_0074817 F | GGTGTTGTGGAAGTCACACTG |
| hsa_circ_0074817 R | TCCATCCTTCACTCGGGCT |
| PKNOX1 F | CCAAACTGCTCTGAACCCGA |
| PKNOX1 R | CCTTCAGAGCCCTGTGTAGAT |
| hsa-miR-1208 | CTCACTGTTCAGACAGGCGGA |
| hsa-miR-1226-5p | GTGAGGGCATGCAGGCC |
| hsa-miR-1229-5p | GTGGGTAGGGTTTGGGGGA |
| hsa-miR-370-3p | GCCTGCTGGGGTGGAAC |
| hsa-miR-432-5p | CTCTTGGAGTAGGTCATTGGGTGG |
| hsa-miR-4656 | TATGGGCTGAGGGCAGGAG |
| hsa-miR-4686 | CTATCTGCTGGGCTTTCTGGTGTT |
| hsa-miR-4701-3p | ATGGGTGATGGGTGTGGTGT |
| hsa-miR-4715-3p | GTGCCACCTTAACTGCAGCCAAT |
| hsa-miR-4721 | TGAGGGCTCCAGGTGACG |
| hsa-miR-4722-3p | TACCTGCCAGCACCTCCC |
| hsa-miR-4793-3p | TCTGCACTGTGAGTTGGCTGG |
| hsa-miR-550a-5p | TAGTGCCTGAGGGAGTAAGAGCC |
| hsa-miR-6071 | TTCTGCTGCCGGCCAAG |
| hsa-miR-670-5p | CCGTCCCTGAGTGTATGTGGTG |
| hsa-miR-6757-5p | TAGGGATGGGAGGCCAGGAT |
| hsa-miR-6785-5p | TGGGAGGGCGTGGATGATG |
| hsa-miR-6786-3p | TGACGCCCCTTCTGATTCTGC |
| hsa-miR-6794-5p | TATACAGGGGGACTGGGGGT |
| hsa-miR-6798-5p | TATACCAGGGGGATGGGCGA |
| hsa-miR-6811-3p | TAGCCTGTGCTTGTCCCTGC |
| PRPF38A-F | CCATGGACCAACCAAGCTGT |
| PRPF38A-R | TATTGGGCTTTGTGCGTGGG |
| KCTD15-F | CCAACGATACTCTGGGCAGG |
| KCTD15-R | ATTGGACTTGGTGAGCTGGG |
| RAB7A-F | CAGACTGCTGCGTTCTGGTA |
| RAB7A-R | GCTTTGTGGCCACTTGTCTG |
| HSP90AB1-F | CGGCTGTACTGTGCTTCGC |
| HSP90AB1-R | TGCACTTCCTCAGGCATCTAA |
| HMGA2-F | CAGCAAGAACCAACCGGTGA |
| HMGA2-R | AAGGCAACATTGACCTGAGC |
| RAD54L2-F | GCAGGAGCACTGTGGCTATTTG |
| RAD54L2-R | GCAGACACTCCCAGCATGTAT |
| DCK-F | AAAGTCAAACCCCGACACCC |
| DCK-R | GGGGTGGCCATTCCTTAGTC |
| BAG4-F | GGAGGAAGCCACCAGAGTTT |
| BAG4-R | TGGGCTGTTGCCAGATGAAC |
| TNRC6B-F | ATGAAAGCAGTCCTGGGTGG |
| TNRC6B-R | CCATCCTCCTCCTCTTCCCA |
| CYB561D1-F | TGGAGGTAGGTCTGGTTCCC |
| CYB561D1-R | TGAAGCCCAAAGCTACCAGG |
| RETSAT-F | CTGGATGTGGTGGTAATTGGC |
| RETSAT-R | GGTATGTTGTTCCAGCACCAG |
| FOXO1-F | CCACATTCAACAGGCAGCAG |
| FOXO1-R | CCATCCACATCGAGGCTCC |
| CANX-F | GCCTCCGCCTCTCTCTTTAC |
| CANX-R | GGAGGAGCAGTGGTATCTGG |
| NSUN4-F | TTCTTCTATGACCGGCACGC |
| NSUN4-R | CGGCAGTAGGTCCTTTCCAG |
| NFASC-F | GGCTGGTCTCTGCCCTAATG |
| NFASC-R | AATCAACCTCCAAGGGCTCG |
| LIN28A-F | TGGATGGATCAAACCTCCTTAATTT |
| LIN28A-R | TATTCCCTTTCTTGGCCTCCTG |
| DHX33-F | GGTCCTCATCGGTTGGCTAT |
| DHX33-R | TCCGTTCGTGAGCTTCATCC |
| PARVB-F | TGTGCACAAATGCACCATGT |
| PARVB-R | GAACCAGCCAGAGAAGCCAT |
| TGFBR2-F | TTGGCGAGGAGTTTCCTGTT |
| TGFBR2-R | GAGGGAAGCTGCACAGGAGT |
| KIAA0355-F | GCGTCGCAGGAAAGGACTTA |
| KIAA0355-R | GCTGAGGTAGCGGTTTCCTT |
| ARID3B-F | GGCAGAAGACAGAGCAGAGG |
| ARID3B-R | CTTCTGGGCAAACAGCACAC |
| NF1-F | AAACCAGCGGAACCTCCTTC |
| NF1-R | CTGGCTAACCACCTGGTATAAA |
| MGMT-F | ACCGTTTGCGACTTGGTACT |
| MGMT-R | TGCTCACAACCAGACAGCTC |
