# Supplementary material for: Inhibition of intervertebral disc disease progression via the circPKNOX1–miR-370-3p–KIAA0355 axis
Source: Cell Death Discov. 2021 Feb 26;7:39. doi: 10.1038/s41420-021-00420-4 (PMC7910476; doi:10.1038/s41420-021-00420-4)
Supplement: Supplementary file 2 — Supplementary Figure Legends.docx [file 41420_2021_420_MOESM2_ESM.docx]

**Figure S3：**

(**Figure S3**) RT-qPCR was used to detect the expression levels of hsa_circ_0074817, hsa_circ_0000720, hsa_circ_0007018, hsa_circ_0000357, and hsa_circ_0061853. (*p < 0.05. Data represent mean ± S.D., and the P values were determined by a two-tailed unpaired Student’s t-test.).
