# Supplementary figures and images for: Inhibition of intervertebral disc disease progression via the circPKNOX1–miR-370-3p–KIAA0355 axis
Source: Cell Death Discov. 2021 Feb 26;7:39. doi: 10.1038/s41420-021-00420-4 (PMC7910476; doi:10.1038/s41420-021-00420-4)

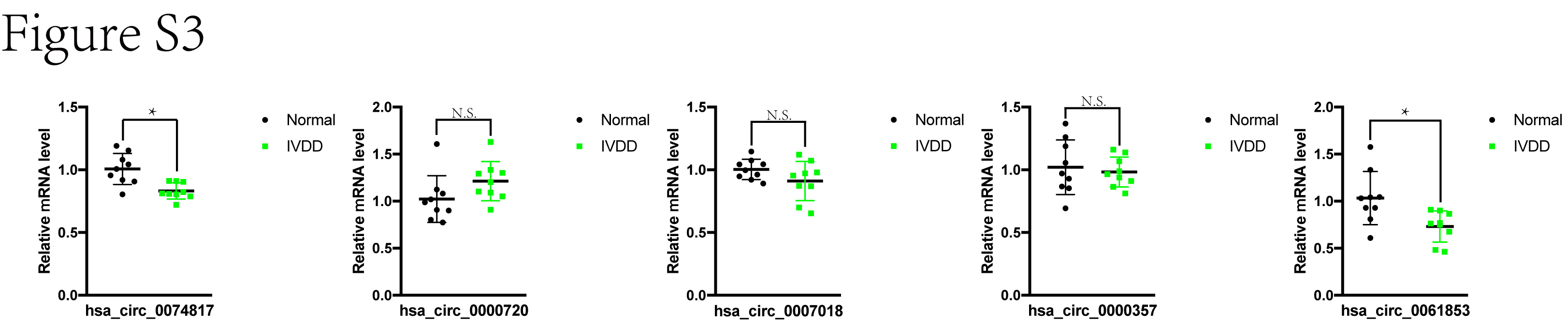

Supplement: Supplementary file 3 — Supplementary Figure Legends.tif [file 41420_2021_420_MOESM3_ESM.tif]
